# Linking Ecological Stoichiometry to Biomass Allocation in Plants Under Cadmium and Petroleum Stress in the Yellow River Delta

**DOI:** 10.3390/biology14060673

**Published:** 2025-06-10

**Authors:** Shuo Li, Haidong Xu, Hui Ye, Cheng Chang, Jinxiang Zhao, Jiangbao Xia

**Affiliations:** 1 Shandong Key Laboratory of Eco-Environmental Science for the Yellow River Delta, Shandong University of Aeronautics, Binzhou 256603, China; 2 Binzhou Hydrographic Bureau, Binzhou 256609, China

**Keywords:** *Suaeda salsa*, pollution stress, stoichiometric characteristics, biomass allocation

## Abstract

Cadmium and petroleum are the main pollutants in coastal wetland ecosystems that affect plant nutrient balance and growth. Scholars have discovered how saline plants adapt to single stresses. How plant ecology and physiology correspond to complex cadmium and petroleum pollution, especially regarding the pollution impacts on carbon (C), nitrogen (N), and phosphorus (P) stoichiometry and biomass allocation in coastal wetland plants, remains unclear, limiting their application in regard to pollution remediation. This study focuses on *Suaeda salsa*, a popular species used in vegetation restoration in the Yellow River Delta’s coastal wetlands. Through the use of pot experiments, the dynamic changes in plant–soil ecological stoichiometry and biomass allocation were systematically investigated in response to individual and combined cadmium (0, 5, and 10 mg kg^−1^) and petroleum (0, 6, and 12 g kg^−1^) treatments. Compared with the control (CK), petroleum stress significantly increased the total nitrogen (TN) and plant total phosphorus (TP) contents, but did not substantially impact the total carbon (TC) content, resulting in 19.7% and 26.6% decreases in the plant C/N and C/P ratios, respectively. The soil organic carbon (SOC) content increased significantly under petroleum stress, whereas the TN and TP contents did not notably change, considerably increasing the soil C/N and C/P ratios, which were 1.5 times and 1.3 times greater than those of the CK, respectively. Cadmium stress alone or with petroleum stress did not significantly affect the C, N, or P stoichiometry or biomass allocation in *S. salsa*. The soil C/N/P stoichiometry redundancy analysis revealed that the contribution rates (especially the soil C/P and C/N ratios) to the total biomass and its allocation in *S. salsa* (64.5%) were greater than those of the control group plants (35.5%). The correlation analysis revealed that the total growth biomass of *S. salsa* was negatively correlated with the soil carbon content, C/N ratio, and C/P ratio, but positively correlated with the plant C/N and C/P ratios. The aboveground biomass proportion in *S. salsa* was significantly negatively correlated with the soil N/P ratio. The belowground biomass proportion exhibited the opposite trend. Petroleum pollution was the main factor driving *S. salsa* stoichiometry and growth changes, increasing the soil C/N and C/P ratios, reducing the nitrogen and phosphorus nutrient absorption capacities in plant roots, limiting plant nitrogen and phosphorus nutrients, and inhibiting biomass accumulation. Appropriate N and P supplementation alleviated plant growth inhibition due to petroleum pollution stress, which was conducive to improving vegetation ecological restoration in the Yellow River Delta.

## Simple Summary

Cadmium and petroleum contamination are among the major threats to the wetland ecosystem in the Yellow River Delta. Therefore, understanding how plants grow and regulate nutrient uptake under such pollution stress is essential for effective ecological restoration. Most scientists working in this field aim to understand how plants adjust the balance of carbon, nitrogen, and phosphorus to optimize biomass allocation and enhance the remediation potential in complex environments. Here, we provide evidence that petroleum pollution is the primary driver of changes in the stoichiometric characteristics and reductions in the biomass of halophytic plants (*Suaeda salsa*). Firstly, petroleum contamination significantly increases the soil carbon-to-nitrogen ratio and carbon-to-phosphorus ratio, thereby suppressing plant nutrient uptake. Secondly, this nutrient limitation leads to a decline in total biomass accumulation. As soil stoichiometric ratios play a dominant role in plant growth, appropriate supplementation with nitrogen and phosphorus can mitigate the adverse effects of pollution stress and improve restoration outcomes. Our findings have important implications for vegetation rehabilitation in the Yellow River Delta and provide a theoretical basis for the management of contaminated soils.

## 1. Introduction

Carbon (C), nitrogen (N), and phosphorus (P) are fundamental elements governing plant growth and soil nutrient cycling, with their stoichiometric characteristics serving as key indicators of ecosystem material metabolism and energy flow [1,2]. The adaptability of plants to environmental stress is largely determined by their ability to optimize nutrient use efficiency through the regulation of intracellular C/N/P stoichiometry [3,4]; C/N and C/P ratios indicate the plant’s capacity for carbon assimilation and their potential for growth. Extensive research has established that plant C/N ratios not only represent their carbon fixation efficiency, but also strongly correlate with soil nitrogen availability [5], whereas the N/P ratio serves as a reliable diagnostic parameter for identifying nutrient limitations in plants [6]. Similarly, soil C/N/P stoichiometry provides critical guidance for assessing nutrient constraints, particularly when considering that elevated soil C/N ratios subject to constant organic matter decomposition rates indicate nitrogen limitations [7]; variations in C/P ratios reflect phosphorus dynamics [8], and shifts in N/P ratios correlate with the soil’s carbon content and plant biomass [9]. Under environmental stress, plant-regulated C, N, and P uptake and allocation are critical for sustaining soil nutrient cycling. These processes directly control biomass accumulation patterns [10]. This profound interconnection between elemental stoichiometry and plant resource partitioning underscores the necessity of systematically investigating their relationships, providing a critical theoretical foundation for evaluating vegetation restoration and soil remediation efficacy in the coastal wetlands in the Yellow River Delta.

As the world’s youngest and most intact wetland ecosystem [11], the Yellow River Delta Nature Reserve faces unprecedented environmental challenges, which are driven primarily by the combined stresses of salinization, petroleum pollution, and heavy metal enrichment [12,13,14]. Comprehensive surveys have revealed that this delta contains approximately 180,000 hectares of saline–alkali soils, 28.3% of which are classified as severely affected [12]. Under salt stress, Na^+^ ions have dual detrimental effects, namely direct toxicity and osmotic stress induction [15], which collectively impair plant N and P assimilation and reduce biomass accumulation [16]. Notably, *A. thaliana* maintains osmotic equilibrium by increasing its leaf C/N ratio from 15:1 to >20:1 under high salinity conditions [17]. Concurrently, petroleum pollution from Shengli Oilfield development has exacerbated these challenges [18,19]. Soil monitoring data have indicated that polycyclic aromatic hydrocarbon (PAH) concentrations have reached 47.1 mg kg^−1^ in surface soils [19]. The high viscosity of petroleum hydrocarbons promotes stable exogenous carbon pools, altering the soil’s physicochemical properties and disrupting plant N and P uptake, leading to metabolic dysfunction [20,21]. This contamination process leads to a significant biomass reduction, particularly under combined salt–petroleum stress [22]. In addition, heavy metal pollution is prevalent, with Cd concentrations (0.18 mg kg^−1^) exceeding background levels [14]. Cd competitively inhibits nutrient absorption, disrupting plant–soil stoichiometry. For example, Cd-stressed tomato seedlings exhibit ion efflux and wilting [23]. Similarly, *S. salsa* displays a 29.4–62.5% decline in biomass, but adapts through belowground biomass proportion adjustments [24].

As key species for coastal wetland remediation, representative plants from the Poaceae (e.g., common reed, green bristlegrass, and tall fescue), Fabaceae (e.g., alfalfa), and Amaranthaceae (e.g., *S. salsa*) families demonstrate remarkable remediation potential as a result of their unique biomass allocation and C/N/P stoichiometry [25,26,27]. In regard to remediation practices in the Yellow River Delta, particular preference is given to halophytes that have both a salt tolerance and strong contaminant accumulation capacity, including *S. salsa*, common reed, and tamarisk [28,29,30]. In particular, *S. salsa*, a pioneer species in coastal wetlands, exhibits exceptional stress resistance and can survive when subject to multiple environmental stressors [31,32,33,34]. Experimental data have demonstrated that planting *S. salsa* in petroleum-contaminated areas results in 40–60% degradation of PAHs, while increasing the soil organic matter content by 15–25% and enhancing the vegetation coverage from <10% to 50% [35]. Moreover, this species shows distinctive Cd accumulation characteristics. Under a Cd stress of 15 mg kg^−1^, the aboveground Cd content of *S. salsa* reaches 52.16 mg kg^−1^, with its concentration in the roots reaching 98.53 mg kg^−1^, and an excellent accumulation capacity of 2.30 g per plant [36]. These findings establish *S. salsa* as the species of choice for the remediation of moderately to highly Cd-contaminated sites. However, the mechanisms by which the tripartite interactions of salinity, Cd, and petroleum contamination dynamically regulate plant–soil C/N/P stoichiometry are poorly understood and warrant further investigation. The stress tolerance of *S. salsa* likely stems from its adaptive biomass allocation strategies, coupled with the dynamic regulation of C, N, and P nutrients, which are closely associated with the partitioning and uptake of soil nutrients [37]. Nevertheless, the mechanistic interactions between combined Cd and petroleum contamination under moderate salinity conditions have yet to be systematically elucidated.

To highlight the underlying tolerance mechanisms of *S. salsa* to cadmium and petroleum stress and its impact on soil physicochemical properties, a pot experiment was conducted. The experimental design incorporated factorial gradients of Cd (0, 5, and 10 mg kg^−1^) and petroleum (0, 6, and 12 g kg^−1^) to systematically investigate the regulatory effects of multiple stressors on the stoichiometric characteristics and nutrient cycling in regard to the plant–soil system. Specifically, we present a study focused on two key aspects. First, the effects of cadmium and petroleum stress on the C, N, and P contents and their stoichiometric ratios in *S. salsa* plant tissues and soil were investigated. Second, correlation analyses between the biomass allocation indices in *S. salsa* and plant–soil C/N/P stoichiometric characteristics and soil environmental factors (e.g., AP, NO_3_^−^, NH_4_^+^, Cd, and petroleum), were performed to reveal the linkages between nutrient cycling and plant adaptation under the combined stress of Cd and petroleum contamination.

## 2. Results

### 2.1. Effects of Cd and Petroleum Stress on Soil Physicochemical Properties

The soil NO_3_^−^ content was significantly affected by the individual Cd treatments (i.e., Cd_1_P_0_ and Cd_2_P_0_). However, the TC, AP, and NH_4_^+^ contents did not significantly change as a result of these treatments. The soil NO_3_^−^ content exhibited a unimodal response pattern, initially increasing with an increasing Cd concentration and subsequently decreasing. Compared with that of the CK group, the NO_3_^−^ content in the Cd_1_P_0_ treatment group increased by 23.35%, whereas it decreased by 22.57% in the Cd_2_P_0_ treatment group. The soil TC, AP, and NO_3_^−^ contents were significantly influenced by the single petroleum treatments (i.e., Cd_0_P_1_ and Cd_0_P_2_) (*p* < 0.01), whereas the NH_4_^+^ content remained unaffected by the single petroleum treatments (*p* > 0.05) (Table 1). The increase in the TC content under petroleum stress was concentration dependent, with the Cd_0_P_2_ treatment group showing a 34.63% increase in the TC content relative to that of the CK group (*p* < 0.01) (Table 1). In contrast, both the AP and NO_3_^−^ concentrations decreased significantly with an increasing petroleum concentration (*p* < 0.01) (Table 1). Compared with the CK group, the AP and NO_3_^−^ concentrations were reduced by 25.61% and 57.98%, respectively, in the Cd_0_P_2_ treatment group. The AP content (*p* < 0.05) and NO_3_^−^ content (*p* < 0.01) in the soil were significantly influenced by a combination of Cd and petroleum stress (Table 1). Under all the combined stress treatments, the soil AP and NO_3_^−^ contents were lower than the CK group. Compared with the Cd_1_P_0_ treatment, the Cd_1_P_1_ treatment significantly increased the AP content by 13.64% (*p* < 0.05) (Table 1). Compared with the Cd_0_P_2_ treatment, the Cd_1_P_2_ and Cd_2_P_2_ treatments significantly decreased the NO_3_^−^ content (*p* < 0.05), and the Cd_2_P_2_ treatment group presented the lowest NO_3_^−^ content, which was 64.20% lower than the CK group (Table 1).

### 2.2. Effects of Cd and Petroleum Stress on the Plant Biomass Allocation Ratio

**Figure 1:**
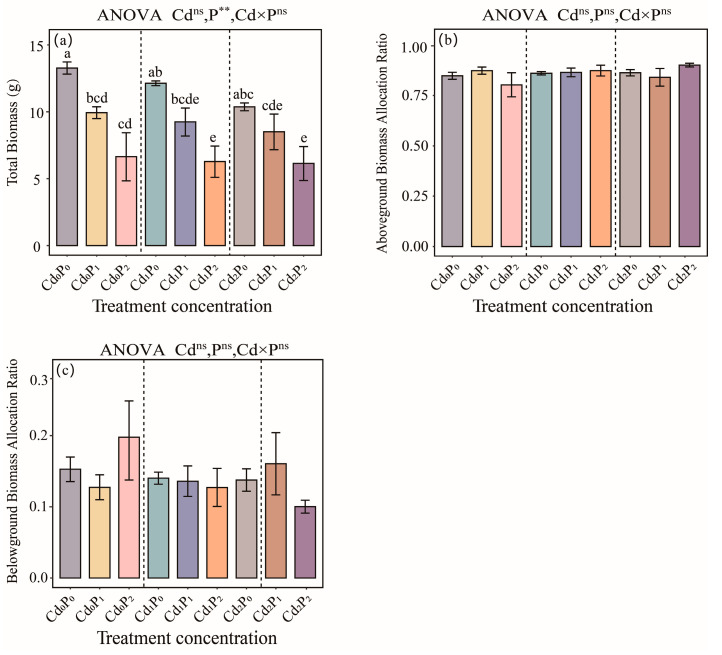
Effects of single Cd, single petroleum, and combined Cd–petroleum treatments on *Suaeda salsa* biomass parameters, including (**a**) total biomass, (**b**) aboveground biomass allocation ratio, and (**c**) belowground biomass allocation ratio. Different lowercase letters denote significant differences between various treatments (*p* < 0.05), and there were no differences between the unlabeled groups. **, *p* < 0.01; ns, *p* > 0.05. The same applies below.

A decreasing trend in the TB was observed in *S. salsa* as a result of the single Cd treatment, single petroleum treatment, and combined Cd–petroleum treatment (Figure 1). Notably, the single petroleum treatment had a highly significant effect on the TB of *S. salsa* (*p* < 0.01, Figure 1a), with the Cd_2_P_2_ treatment group showing the lowest TB (6.13 g), corresponding to a 63.0% reduction compared with that of the CK. However, there were no significant effects of the different treatments on the AGBP or BGBP (*p* > 0.05, Figure 1b,c). The minimum AGBP occurred in the Cd_0_P_2_ treatment group, representing a 5.29% decrease relative to that of the CK group (*p* > 0.05, Figure 1b), whereas a high BGBP was observed in the Cd_0_P_2_ and Cd_2_P_1_ treatment groups, with values that were 29.4% and 5.0% greater than those of the CK group, respectively. In contrast, the Cd_2_P_2_ treatment group presented the lowest BGBP, with a 34.4% reduction relative to that of the CK group (*p* > 0.05, Figure 1c).

### 2.3. Effects of Cd and Petroleum Stress on the Stoichiometry of Suaeda salsa

**Figure 2:**
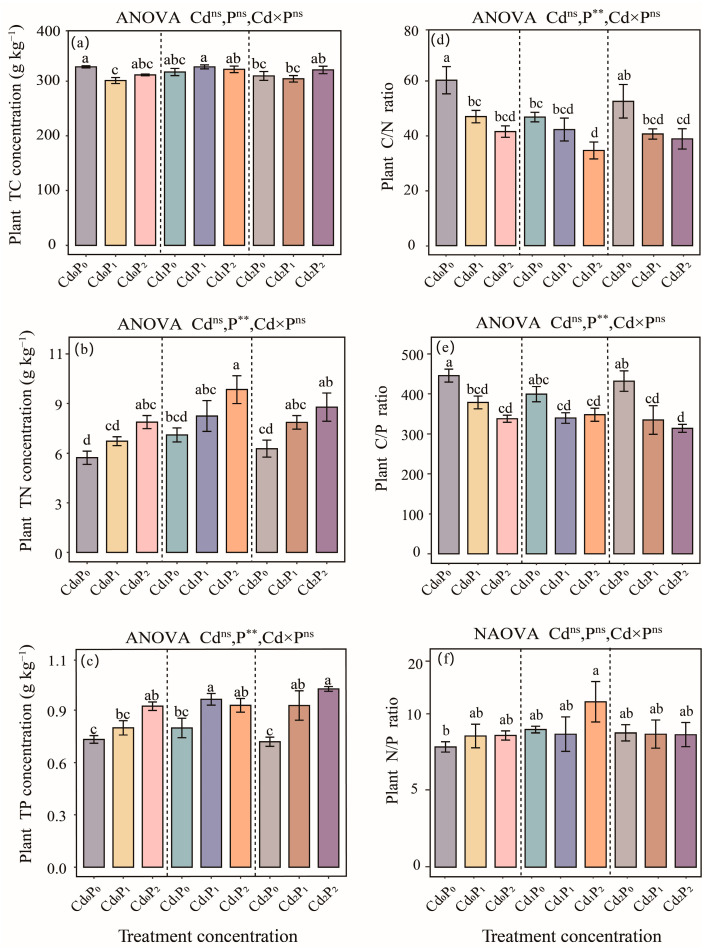
Effects of single Cd, single petroleum, and combined Cd–petroleum treatments on the ecological stoichiometry of *Suaeda salsa*, including the (**a**) plant total carbon (C) concentration, (**b**) plant total nitrogen (N) concentration, (**c**) plant total phosphorus (P) concentration, (**d**) plant carbon–nitrogen (C/N) ratio, (**e**) plant carbon–phosphorous (C/P) ratio, and (**f**) plant nitrogen–phosphorous (N/P) ratio. Different lowercase letters denote significant differences between various treatments (*p* < 0.05), and there were no differences between the unlabeled groups. **, *p* < 0.01; ns, *p* > 0.05.

The N content, P content, C/N ratio, and C/P ratio of *S. salsa* were significantly influenced by the single petroleum treatment (*p* < 0.01, Figure 2b–e). A concentration-dependent increase in both the N and P contents was evident with an increasing petroleum concentration (*p* < 0.01, Figure 2b,c). Among the treatments, the highest N content was detected in the Cd_1_P_2_ treatment group, with a significant 70.0% increase compared with that of the CK group (*p* < 0.05, Figure 2b,c). Moreover, the maximum P content was measured in the Cd_2_P_2_ treatment group, which represented a 39.5% increase compared with that of the CK group, whereas the Cd_2_P_0_ treatment group presented the lowest value, with a 2.27% decrease relative to that of the CK group (*p* < 0.01, Figure 2c). Under combined Cd–petroleum stress, both the C/N and C/P ratios of *S. salsa* were significantly lower than the CK group (*p* < 0.01, Figure 2d,e), although the extent of the reduction varied. Compared with the CK group, the plant C/N ratio in the Cd_1_P_2_ treatment group decreased by 42.5% (*p* < 0.01, Figure 2d), whereas the C/P ratio decreased by 29.6% in the Cd_2_P_2_ treatment group (*p* < 0.01, Figure 2e). In contrast, the C content and N/P ratio of *S. salsa* were not affected by the single Cd treatment, single petroleum treatment, or combined stress (*p* > 0.05, Figure 2a,f).

### 2.4. Effects of Cd and Petroleum Stress on Soil Stoichiometry

**Figure 3:**
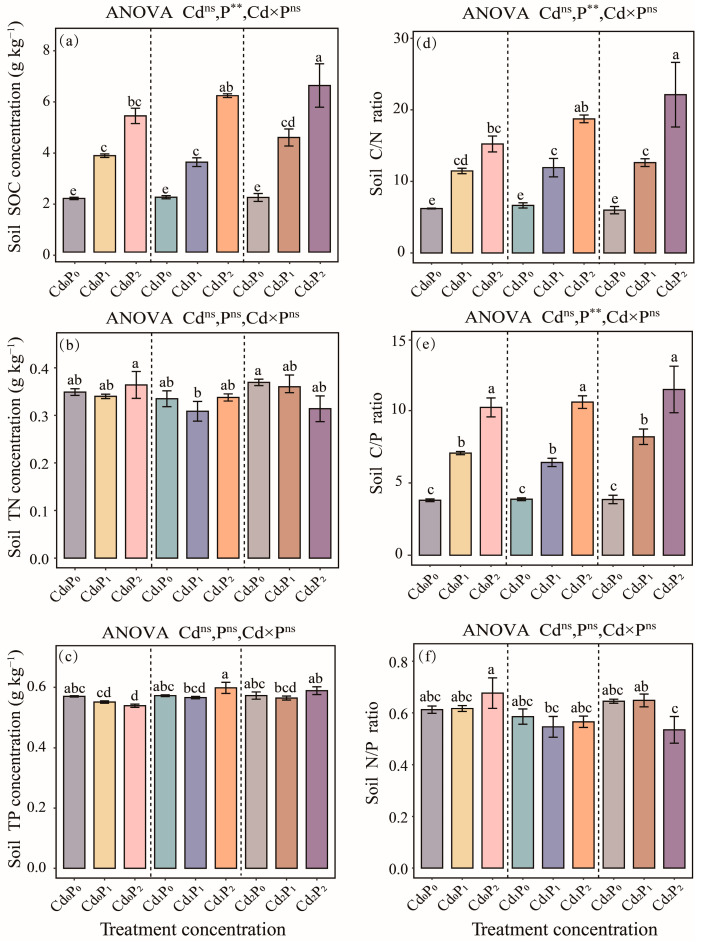
Effects of single Cd, single petroleum, and combined Cd–petroleum treatments on the ecological stoichiometry of soil, including the (**a**) soil organic carbon (SOC) concentration, (**b**) soil total nitrogen (TN) concentration, (**c**) soil total phosphorus (TP) concentration, (**d**) soil carbon–nitrogen (C/N) ratio, (**e**) soil carbon–phosphorous (C/P) ratio, and (**f**) soil nitrogen–phosphorous (N/P) ratio. Different lowercase letters denote significant differences between various treatments (*p* < 0.05), and there were no differences between the unlabeled groups. **, *p* < 0.01; ns, *p* > 0.05.

During the entire stress treatment period, no significant changes were observed in the soil C content, C/N ratio, or C/P ratio under the single Cd treatment (*p* > 0.05, Figure 3a,d,e). However, significant increases in these parameters were induced by all the other treatments (*p* < 0.05, Figure 3a,d,e). Notably, the single petroleum treatment had highly significant effects on the soil C content, C/N ratio, and C/P ratio (*p* < 0.01, Figure 3a,d,e). The most pronounced increases in the C content and C/N ratio were observed in the Cd_2_P_2_ treatment group, with 3.1 and 3.56-fold increases, respectively, compared with the CK group (*p* < 0.05, Figure 3a). Compared with the CK group, the soil C/P ratios significantly increased in the Cd_0_P_2_, Cd_1_P_2_, and Cd_2_P_2_ treatment groups, with 2.6-fold, 2.7-fold, and 3.0-fold increases, respectively (*p* < 0.05; Figure 3e). In contrast, the soil N content, P content, and N/P ratio were not significantly influenced by the single Cd treatment, single petroleum treatment, or their combined application (*p* > 0.05; Figure 3b,c,f). Although the P content decreased by 5.4% in the Cd_0_P_2_ treatment group compared with the CK group, the difference was not statistically significant (*p* > 0.05, Figure 3c).

### 2.5. Correlation Between Biomass Allocation and Chemical Properties in Plant–Soil Systems

**Figure 4:**
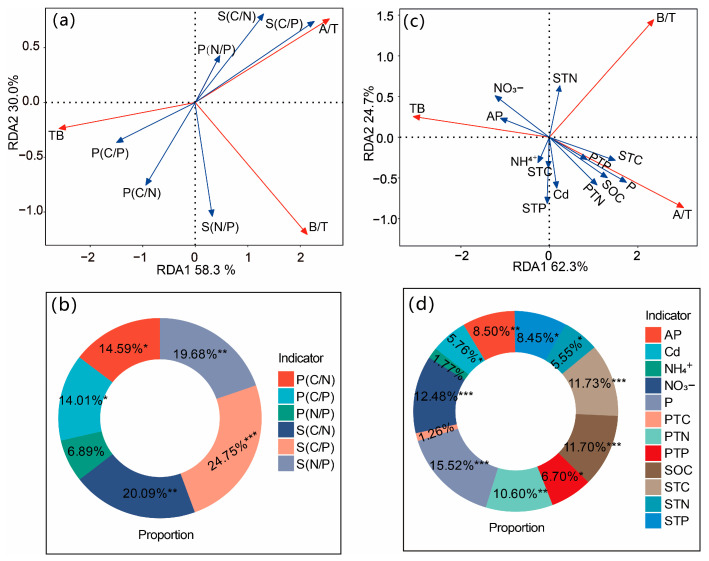
Redundancy analysis (RDA) of total biomass (TB), aboveground biomass proportion (A/Z) and belowground biomass proportion (B/Z) limited by the response ratio of relevant abiotic factors to Cd and petroleum additions (**a**,**c**). The figure shows the percentage change in abiotic factors related to the biomass allocation ratio of *Suaeda salsa* (**b**,**d**). PTC, plant total carbon; PTN, plant total nitrogen; PTP, plant total phosphorus; P (C/N), plant carbon to nitrogen ratio; P (C/P), plant carbon to phosphorus ratio; P (N/P), plant nitrogen to phosphorus ratio; STC, soil total carbon; SOC, soil organic carbon; STN, soil total nitrogen; STP, soil total phosphorus; S (C/N), soil carbon to nitrogen ratio; S (C/P), soil carbon to phosphorus ratio; S (N/P), soil nitrogen to phosphorus ratio; AP, soil available phosphorus; NH_4_^+^, soil ammonium nitrogen; NO_3_^−^, soil nitrate nitrogen; Cd, soil Cd content; and P, soil petroleum content. *, *p* < 0.05; **, *p* < 0.01; ***, *p* < 0.001. The same applies below.

The redundancy analysis (RDA) revealed that biotic and abiotic factors explained 58.3% and 30.0%, respectively, of the variation in the biomass allocation indices of *S. salsa* due to divergent plant and petroleum stoichiometry, cumulatively accounting for 88.3% of the total variance (Figure 4a). However, the effects of the stoichiometric shifts in *S. salsa* and the soil stoichiometric indices on the TB, AGBP, and BGBP were heterogeneous (Figure 4b). The soil C/P ratio, soil C/N ratio, and soil N/P ratio emerged as the primary drivers of biomass allocation, with relative contributions of 22.54%, 18.31%, and 17.93%, respectively. In contrast, plant stoichiometry had moderate but distinct effects, wherein the plant C/P ratio and plant N/P ratio contributed 12.77% and 10.74%, respectively. Furthermore, biotic and abiotic factors explained 62.3% and 24.7%, respectively, of the variation in the biomass allocation indices associated with the plant C, N, P, and soil environmental factors, accounting for 87.0% of the explained variance (Figure 4c). Notably, the soil petroleum concentration was identified as the dominant driver of biomass allocation (15.52% relative contribution), followed by soil NO_3_^−^ (12.48%), soil TC (11.73%), SOC (11.70%), and plant TN (10.60%) (Figure 4c).

**Figure 5:**
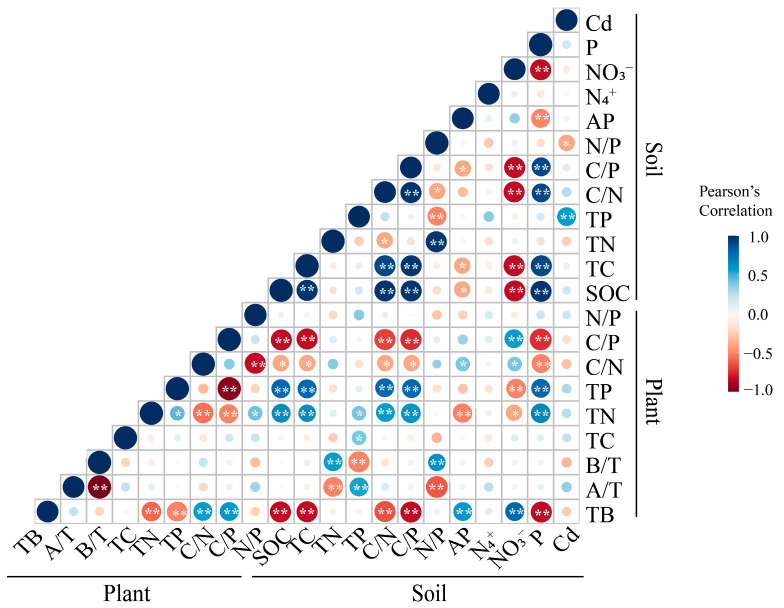
Correlation analysis and heatmap of indicator changes in *Suaeda salsa* under Cd and petroleum stress. *, *p* < 0.05; **, *p* < 0.01.

The TB of *S. salsa* was significantly positively correlated with the soil AP content, soil NO_3_^−^ content, plant C/N ratio, and plant C/P ratio (*p* < 0.01, Figure 5), but significantly negatively correlated with the soil C/N ratio, plant TN content, and plant TP content (*p* < 0.01, Figure 5), as well as with the SOC content, soil TC content, soil C/P ratio, and soil petroleum content (*p* < 0.01, Figure 5). The AGBP of *S. salsa* was significantly positively correlated with the soil TP content (*p* < 0.01, Figure 5), but significantly negatively correlated with the soil TN content and the soil N/P ratio (*p* < 0.01, Figure 5), with no significant correlations observed in regard to the plant C, N, or P contents or ratios (*p* > 0.05, Figure 5). The BGBP of the plants was significantly positively correlated with the soil TN content and the soil N/P ratio (*p* < 0.01, Figure 5), whereas it was significantly negatively correlated with the soil TP content (*p* < 0.01, Figure 5). In contrast, no significant correlations were detected between the plant BGBP and the plant C, N, and P contents or their stoichiometric ratios (*p* > 0.05, Figure 5).

## 3. Discussion

### 3.1. Effects of Cd and Petroleum Stress on Soil Stoichiometry

As a heavy metal with high mobility, Cd^2+^ is prone to competitive adsorption or complexation with NH_4_^+^ and NO_3_^−^ in soil [14,38]. The nonlinear change in the soil NO_3_^−^ under single Cd stress observed in this study is similar to previous results reported by Hu et al. [26]. Notably, low-concentration Cd promotes the conversion of NH_4_^+^ to NO_3_^−^ by changing the characteristics of soil colloids, whereas high-concentration Cd inhibits the absorption of N by plants, leading to a decrease in NO_3_^−^, due to denitrification or leaching after the initial accumulation. This phenomenon reflects the dual regulatory mechanism of Cd in regard to the nitrification process [39]. In the present study, the soil AP content decreases with an increasing Cd concentration, which may have been caused by the precipitation of Cd^2+^ with PO_4_^3−^, as verified in the study by Huang et al. [38]. Hu et al. [26] reported that a high concentration of Cd significantly inhibits NH_4_^+^ adsorption, whereas the single Cd treatment in the present study does not significantly affect the soil NH_4_^+^ content, which may have resulted from salt stress masking the inhibitory effect of Cd on NH_4_^+^ adsorption through competitive adsorption. In regard to the petroleum pollution treatment, the soil TC and SOC contents are positively correlated with the petroleum concentration, whereas the AP, NH_4_^+^, and NO_3_^−^ contents are negatively correlated with the petroleum concentration. This phenomenon may have occurred due to petroleum, an exogenous organic carbon input source, adhering to the surfaces of soil particles because of its high viscosity and low volatility, resulting in carbon accumulation [30,32]. The hydrophobicity of petroleum reduces soil pore connectivity, which not only explains the decreases in the AP and NO_3_^−^ contents, but also explains the increases in the C/N and C/P ratios. Although studies have shown that petroleum limits the release of AP and NO_3_^−^ through physical adsorption, leading to NH_4_^+^ accumulation [30], we did observe a decrease in the NH_4_^+^ content in this study, possibly because *S. salsa* enhances N uptake to maintain normal physiological growth. The “carbon enrichment–nutrient depletion” model proposed by Lerdau et al. [40] reveals the decoupling between C metabolism and nutrient cycling under petroleum pollution. Notably, the inhibitory effect of combined Cd and petroleum stress on AP and NO_3_^−^ is significantly greater than that of single stress. This finding aligns with the results reported by Xiao et al. [27], who reported P fixation under Cu–petroleum combined contamination conditions. However, unlike the results for Cu–petroleum combined contamination, the C/N ratio change as a result of the Cd–petroleum treatment presents unique characteristics, which may be related to the carbon-dominant effect of petroleum input.

The stoichiometric characteristics of soil C/N/P ratios serve as key indicators for evaluating soil nutrient cycling and ecological functions [41], and their change patterns are regulated by multiple factors. The soil C/N ratio reflects the potential for organic matter decomposition and nitrogen mineralization. In this study, the measurement reached 43.0, which is significantly greater than the regional average of 30.4 for eastern China’s coastal wetlands [25]. This discrepancy can be attributed to the following factors: first, the inherently low background nitrogen content in Yellow River Delta soils has been well-documented [37], and, second, the exogenous carbon input from petroleum contamination increases the C/N ratio. Notably, the soil C/P ratio, a key indicator of the phosphorus mineralization capacity [27], reached 370.3 this study, which is far above the average of 31.4 in the region. This extreme increase can be attributed to the synergistic effects of the physical coating by hydrophobic petroleum compounds and the formation of Cd_3_(PO_4_)_2_ precipitates, both of which contribute to phosphorus fixation. Comparable evidence is provided by the soil N/P ratio (0.6), which is not only lower than the regional average (2.1), but is also in sharply contrast with that of plantation systems (5.1) [42], indicating that the relative nitrogen deficiency resulting from *S. salsa* prefers a nitrogen retention strategy under combined stress. Although Xiong et al. [25] have demonstrated the importance of regional variation and the growing season on soil element dynamics, our results highlight how the synergistic effects of moderate salinity, combined Cd–petroleum contamination, and *S. salsa* cultivation collectively drive the observed stoichiometric shifts, increase the C/N ratio, increase the C/P ratio, and decrease the N/P ratio.

### 3.2. Effects of Cadmium and Petroleum Stress on the Biomass Allocation and Elemental Stoichiometry of Suaeda salsa

Cd pollution significantly inhibits plant root growth and nutrient absorption [43], whereas petroleum hydrocarbons primarily hinder C transportation via physical barriers, without significantly affecting C accumulation in plants [44]. The current research shows that petroleum contamination alone does not significantly influence the C content of *S. salsa*, suggesting that this halophyte might maintain C homeostasis at specific pollution levels through physiological regulation. Notably, the N and P contents increase with an increasing petroleum concentration, likely because petroleum hydrophobicity reduces soil porosity, while increasing NO_3_^−^ availability and P solubility, thereby promoting root nutrient acquisition for stress resistance. These results are aligned with prior findings. Under combined Cd–petroleum stress, the TB, C/N, and C/P ratios of *S. salsa* decreased, which is consistent with the carbon–nutrient balance hypothesis proposed by Lerdau et al. [40]. Plants allocate resources to secondary metabolites as per stoichiometric demand, leading to reduced structural C/N/P ratios and inhibited biomass accumulation. In the present study, under the combined stress of Cd and petroleum, the overall trend in the AGBP and BGBP allocation in *S. salsa* is not significant. However, under high-concentration petroleum stress alone, the increase in the BGBP is the most pronounced. This adaptive strategy may compensate for the reduced nutrient uptake efficiency by expanding the absorptive surface area. As a critical indicator of nutritional status, C/N/P stoichiometry is a critical indicator of environmental adaptation strategies. Compared with the natural *S. salsa* stoichiometry (C/N = 20.1, C/P = 253.3, and N/P = 15.3) reported by Zhao et al. [31], we have obtained higher C/N (41.1) and C/P (360.5) ratios and lower N/P (8.89) ratios, reflecting the enhanced N and P uptake under multiple stress conditions. Following Koerselman and Meuleman’s criteria (N/P < 14 indicates N limitation) [6], the present study confirms N as the primary limiting factor, aligning with typical halophyte adaptations in N-deficient environments. Overall, petroleum pollution in saline habitats triggers distinct C/N/P imbalances in *S. salsa*, underscoring site-specific N and P fertilization to alleviate growth constraints, which are dynamically adjusted to salinity and pollution gradients.

### 3.3. Relationships Between Plant–Soil C, N, and P Concentrations and Stoichiometry and Their Responses to Plant Biomass

The allocation of plant biomass, including shifts in the distribution of aboveground and belowground biomass, significantly influences ecosystem functions. Studies have demonstrated that plant biomass allocation is regulated by soil nutrient limitations and is further modulated by the C/N stoichiometry within plant tissues [41]. In the present study, compared with the intrinsic stoichiometric characteristics of plants, the stoichiometric properties of the soil C/N/P ratio contribute more substantially to the total biomass and its allocation in saline land. Among these factors, the TB of *S. salsa* is significantly correlated with the soil’s available phosphorus (AP), NO_3_^−^, total organic carbon (SOC), total carbon (TC), C/N ratio, C/P ratio, plant TP, TN, C/N ratio, and C/P ratio. Additionally, the AGBP and BGBP are significantly correlated with the soil TP content, TN content, and N/P ratio, highlighting the critical role of the soil nutrient supply in plant growth. Notably, the soil C/N and C/P ratios have the most pronounced effects on *S. salsa* growth [34]. Among these factors, the soil NO_3_^−^ content has the most substantial impact on the plant biomass allocation ratio, further confirming that nitrogen availability is the primary constraint on plant growth. This finding aligns with previous research results. Scholars have indicated that in stressful environments, where soil nutrients are unevenly distributed, NO_3_^−^ becomes the preferred nitrogen source for plants because of its high solubility and ease of migration [45]. Furthermore, we have revealed that the TB of saline land is negatively associated with the soil C content, which can be attributed to the increased amounts of N and P that are transformed into soluble forms (e.g., NH_4_^+^ and NO_3_^−^) during SOC decomposition. This process increases plant demand for N and P nutrients. Research has shown that in N-limited environments, soil C/N ratios are typically elevated, necessitating increased N-use efficiency in plants to sustain growth [31]. Further analysis indicates that TB is negatively correlated with the soil C content, C/N ratio, and C/P ratio, but positively correlated with plant TN content, TP content, C/N ratio, and C/P ratio. This phenomenon may be explained by the accumulation of SOC, which reduces N and P availability and disrupts the C/N/P stoichiometric balance, thereby inhibiting TB accumulation. Additionally, the AGBP is negatively correlated with the soil TN, positively correlated with the soil TP, and exhibits a significantly negative correlation with the soil N/P ratio, whereas the BGBP exhibits an inverse pattern. This observation suggests that plants adapt to environmental stress by regulating the C/N and C/P ratios, thereby optimizing growth and nutrient uptake efficiency. The equilibrium in terms of the soil C/P and N/P ratios serves as an indicator of P usability. When the soil P content is low, plants modify the aboveground and belowground biomass allocation patterns to optimize P absorption [41,46]. We have shown that plant biomass is more strongly influenced by soil nutrient conditions than by its own stoichiometric characteristics, with the C/N and C/P ratios exerting the most significant regulatory effects on plant growth. Moreover, the present study highlights the dominant role of C/N/P ecological stoichiometry in regulating *S. salsa* biomass and the impact of C/N/P coupling effects on plant biomass distribution.

### 3.4. Future Prospects

In the current research, we offer a comprehensive examination of the effects of cadmium and petroleum contamination on soil stoichiometric ratios and the growth of salt-tolerant plants (*S. salsa*), revealing the complex mechanisms through which pollutants influence soil nutrient cycling and plant nutrient uptake. These findings offer valuable data support and a theoretical foundation for the rehabilitation of polluted soils and for the maintenance of the plant–soil stoichiometric balance. However, this study has certain limitations and shortcomings. Although the combined effects of cadmium and petroleum pollution have been considered, other environmental factors that may influence soil and plant stoichiometric ratios, such as alterations in soil microbial communities and enzyme activity, have not been fully examined. Additionally, beyond the C, N, and P stoichiometric ratios in plant–soil systems, microbial stoichiometric characteristics can provide thorough information on the disruptive impacts of pollutants on plant physiology and soil ecosystems. Moreover, combined with the analysis of plant physiological indicators, the influence process and mechanism of action are discussed. Future research should investigate pollutant interactions, incorporate various stress-tolerant plant species and different remediation approaches, and assess the effects of cadmium and petroleum contamination on microbial stoichiometry and plant physiology. Long-term field monitoring should help evaluate remediation strategies, providing a scientific basis for sustainable coastal wetland management and vegetation restoration.

## 4. Materials and Methods

### 4.1. Soil and Plants

The soil used in this study was collected from the coastal salt marsh in the Yellow River Delta in Dongying, Shandong Province, China (38°9′51″–38°10′44″ N and 118°2′15″–118°4′32″ E). The soil was characterized as a typical fluvo-aquic soil that was not contaminated by cadmium (Cd) or petroleum. The initial soil properties were 46.58% sand, 47.78% silt, 5.64% clay, pH 7.49, 1.31 g cm−3 soil bulk density, and 0.12% salt content. After the soil used for the pot tests was allowed to air dry at room temperature, it was sieved through 100 mesh holes to remove any remaining plant roots and debris. Sodium chloride (NaCl), Cd chloride (CdCl_2_·2.5H_2_O), and petroleum hydrocarbons (PHCs) were added to obtain the target Cd and petroleum concentrations.

**Figure 6:**
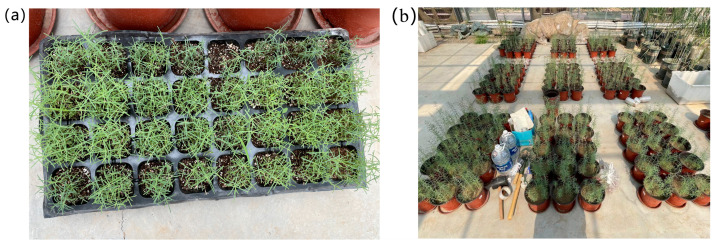
Experimental assembly showing *Suaeda salsa* plants grown in pots in a greenhouse. Seed germination (**a**); seedling transplantation (**b**).

*Suaeda salsa* (ecotype: saline–alkali) seeds were collected from the coastal tidal flat in the Yellow River Delta in November 2023 and were naturally air dried, before being stored in a refrigerator at 4 °C. The seeds were disinfected with a 5% hydrogen peroxide solution for 15 min, rinsed with sterile water, and soaked for a period of 24 h. Subsequently, the disinfected seeds were sown in a large plastic tray (Figure 6a). After 10 days of germination, healthy and uniformly sized seedlings were selected and transplanted into pots measuring 180 × 140 mm (10 seedlings per pot), which were filled with 1.5 kg of the experimental soil (Figure 6b). Two layers of mesh were placed at the bottom, and each treatment was replicated five times, under consistent experimental conditions.

### 4.2. Pot Experiment

The experiment comprised nine treatments, based on varying levels of Cd and petroleum contamination. The Cd concentrations used were 0 mg kg^−1^ (control, C_0_), 5 mg kg^−1^ (low, C_1_), and 10 mg kg^−1^ (high, C_2_), whereas the petroleum concentrations used were 0 g kg^−1^ (control, P_0_), 6 g kg^−1^ (low, P_1_), and 12 g kg^−1^ (high, P_2_). The treatments were as follows: a control group without Cd or petroleum (C_0_P_0_); petroleum-only treatments with low (C_0_P_1_) and high (C_0_P_2_) concentrations; Cd-only treatments with low (C_1_P_0_) and high (C_2_P_0_) concentrations; and combined Cd and petroleum treatments, including low Cd with low petroleum (C_1_P_1_), low Cd with high petroleum (C_1_P_2_), high Cd with low petroleum (C_2_P_1_), and high Cd with high petroleum (C_2_P_2_) concentrations. This design allowed for the evaluation of the individual and interactive effects of Cd and petroleum contamination on soil conditions and plant responses [47,48]. The application levels of Cd and petroleum were determined based on the National Soil Environmental Quality Standard of China [49], the guidelines from the Ministry of Environmental Protection of China [50]), and the toxicity thresholds for *S. salsa* growth [51,52].

The pot experiment was carried out in the greenhouse as part of the Yellow River Delta Ecological Environment Key Laboratory (Dongying, Shandong, China), with a temperature of 26 ± 2 °C, a photoperiod of 16 h, and a relative humidity of 65 ± 5%. Each pot was equipped with a plastic tray to prevent potential petroleum leakage and was filled with 1.5 kg of spiked soil [53]. The plants were irrigated regularly with deionized water during plant growth, using the same nutrient supply and management methods.

### 4.3. Determination of Plant Physiological Characteristics

*S. salsa* plants were grown for 90 days. Following this period, the aboveground and belowground plant parts were first dehydrated at 105 °C for 30 min and, subsequently, dried at 75 °C, until a constant weight was achieved. The dry weights of the aboveground biomass (AGB) and belowground biomass (BGB) were measured with an analytical balance (minimum precision: 0.1 mg). The total biomass (TB) was calculated as the sum of the AGB and BGB dry weights. The values of the TB, aboveground biomass proportion (AGBP), and belowground biomass proportion (BGBP) were determined using Equations (1)–(3). The dried plant materials (roots, stems, and leaves) were ground into a fine powder (<0.25 mm), using a ball mill. The total carbon (TC) and total nitrogen (TN) contents were determined using an elemental analyzer, whereas the total phosphorus (TP) content was measured using the molybdenum–antimony colorimetric method.

Total biomass (TB) = aboveground biomass (AGB) + belowground biomass (BGB)(1)

Aboveground biomass proportion (AGBP) = AGB/TB(2)

Belowground biomass proportion (BGBP) = BGB/TB(3)

### 4.4. Determination of Soil Index

All the soil samples were sieved through a 2 mm mesh and the roots and other coarse debris were removed. The sieved soil samples were then air dried naturally to determine the soil chemistry. The soil TC, TN, TP, SOC, AP, NH_4_^+^, and NO_3_^−^ contents were determined with reference to soil agrochemical analysis methods [54]. The soil cadmium content was measured using inductively coupled plasma–optical emission spectrometry. The soil petroleum content was quantified using Soxhlet extraction, with dichloromethane, followed by gravimetric analysis.

## 5. Conclusions

Cadmium and petroleum stress have divergent effects on *S. salsa* stoichiometry and growth, with only petroleum stress alone causing significant changes in the stoichiometry and growth traits of this species. Specifically, single petroleum stress significantly reduces the C/N and C/P ratios in plants, but increases the C/N and C/P ratios in soils. Redundancy analysis (RDA) has revealed that the contribution rates of the soil C/N/P stoichiometric characteristics to the total biomass and its allocation in *S. salsa* are greater than those of the plants themselves. Among these factors, the influences of the soil C/N and C/P ratios play leading roles in the growth of *S. salsa*. In addition, correlation analysis reveals that the TB is negatively correlated with the soil C/N and C/P ratios. The increases in the soil C/N and C/P ratios further inhibit the accumulation of biomass in *S. salsa* and affects its normal growth. Therefore, studying the stoichiometric characteristics and growth response of *S. salsa* under pollution stress can help reveal the key mechanisms by which soil C/N/P stoichiometry influences plant growth.

## Figures and Tables

**Table 1:** Physical and chemical properties of soils subjected to Cd and petroleum stress.

Treatment	Soil TC (g kg^−1^)	Soil AP (mg kg^−1^)	Soil NH_4_^+^ (mg kg^−1^)	Soil NO_3_^−^ (mg kg^−1^)	Soil Cd Content (mg kg^−1^)	Soil Petroleum Content (mg kg^−1^)
Cd_0_P_0_	10.57 ± 0.10 Ca	1.64 ± 0.02 Aa	5.26 ± 0.26 Aa	2.57 ± 0.18 Aab	-	-
Cd_0_P_1_	12.2 ± 0.61 Ba	1.38 ± 0.2 Ba	5.14 ± 0.13 Aa	1.61 ± 0.11 Ba	-	464.3 ± 148.3 Ba
Cd_0_P_2_	14.23 ± 0.78 Aa	1.22 ± 0.05 Ba	4.66 ± 0.07 Ba	1.08 ± 0.08 Ca	-	787 ± 111.2A b
Cd_1_P_0_	10.8 ± 0.18 Ca	1.32 ± 0.07 Ab	5.05 ± 0.12Aa	3.17 ± 0.51 Aa	4.77 ± 0.64 Ab	-
Cd_1_P_1_	12.02 ± 0.18 Ba	1.5 ± 0.12 Aa	5.27 ± 0.43 Aa	1.69 ± 0.06 Ba	3.60 ± 0.64 Aa	524.6 ± 50.52 Ba
Cd_1_P_2_	14.69 ± 0.26 Aa	1.32 ± 0.16 Aa	5.19 ± 1.05 Aa	0.94 ± 0.04 Cb	3.77 ± 0.59 Ab	996.3 ± 55.16 Aa
Cd_2_P_0_	10.03 ± 0.34 Bb	1.6 ± 0.2 Aa	5.06 ± 0.11 Aa	1.99 ± 0.34 Ab	5.66 ± 0.21 Ba	-
Cd_2_P_1_	13.3 ± 0.92 Aa	1.35 ± 0.21 Aa	5.24 ± 0.33 Aa	1.53 ± 0.07 Ba	2.02 ± 0.56 Cb	493.3 ± 61.37 Ba
Cd_2_P_2_	14.61 ± 1.78 Aa	1.33 ± 0.24 Aa	5.01 ± 0.41 Aa	0.92 ± 0.02 Cb	8.32 ± 0.62 Aa	1031 ± 90.35 Aa
Cd	ns	ns	ns	**		
P	**	**	ns	**		
Cd × P	ns	*	ns	**		

Note: values (mean ± SE), capital letters indicate differences between different petroleum concentrations at the same Cd concentration, and lowercase letters indicate differences between different Cd concentrations at the same petroleum concentration (*p* < 0.05). Treatments: C_0_, C_1_, and C_2_ represent 0, 5, and 10 mg kg^−1^ of Cd, respectively; P_0_, P_1_, and P_2_ represent 0, 6, and 12 g kg^−1^ of petroleum, respectively; *, *p* < 0.05; **, *p* < 0.01; ns, *p* > 0.05; -, numerous values. The same applies below.

## Data Availability

The original contributions presented in the study are included in the article. Further inquiries can be directed toward the corresponding author.

## Abbreviations

*S. salsa*	*Suaeda salsa*
Cd	Cadmium
TC	Total carbon
TN	Total nitrogen
TP	Total phosphorus
SOC	Soil organic carbon
AP	Available phosphorus
TB	Total biomass
AGBP	Aboveground biomass proportion
BGBP	Belowground biomass proportion

## References

[1] Sardans J., Janssens I.A., Ciais P., Obersteiner M., Peñuelas J. (2021). Recent advances and future research in ecological stoichiometry. Perspect. Plant Ecol. Evol. Syst..

[2] Liu J., Wang Y., Li Y., Zhang Q., Li Z. (2023). Soil ecological stoichiometry synchronously regulates stream nitrogen and phosphorus concentrations and ratios. Catena.

[3] Augusto L., Achat D.L., Jonard M., Vidal D., Ringeval B. (2017). Soil parent material-A major driver of plant nutrient limitations in terrestrial ecosystems. Glob. Change Biol..

[4] Li D., Li Y., Xie Y., Cui B., Ning Z.H., Zhang S.Y., Bi Z.G., Fu S.Q., Che C.G. (2022). Effects of ecological restoration on soil biogenic elements and their ecological stoichiometry in the Yellow River Delta, China. Front. Mar. Sci..

[5] Liu J., Qiu T., Peñuelas J., Sardans J., Tan W., Wei X., Cui Y., Cui Q., Wu C., Liu L. (2023). Crop residue return sustains global soil ecological stoichiometry balance. Glob. Change Biol..

[6] Koerselman W., Meuleman A.F.M. (1996). The vegetation N:P ratio: A new tool to detect the nature of nutrient limitation. J. Appl. Ecol..

[7] Terrer C., Jackson R.B., Prentice I.C., Keenan T.F., Kaiser C., Vicca S., Fisher J.B., Reich P.B., Stocker B.D. (2019). Nitrogen and phosphorus constrain the CO_2_ fertilization of global plant biomass. Nat. Clim. Change.

[8] Sun Y., Wang C., Chen X., Liu S., Lu X., Chen H.Y.H., Ruan H. (2022). Phosphorus additions imbalance terrestrial ecosystem C: N: P stoichiometry. Glob. Change Biol..

[9] Kerkhoff A.J., Enquist B.J., Elser J.J., Fagan W.F. (2005). Plant allometry, stoichiometry and the temperature-dependence of primary productivity. Glob. Ecol. Biogeogr..

[10] Wang L., Lin G., Li Y., Qu W., Wang Y., Lin Y., Huang Y., Li J., Qian C., Yang G. (2024). Phenotype, Biomass, Carbon and Nitrogen Assimilation, and Antioxidant Response of Rapeseed under Salt Stress. Plants.

[11] Li T., Sun J., Fu Z. (2021). Halophytes Differ in Their Adaptation to Soil Environment in the Yellow River Delta: Effects of Water Source, Soil Depth, and Nutrient Stoichiometry. Front. Plant Sci..

[12] Lim S.J., Shin M.N., Son J.K., Song J.D., Cho K.H., Lee S.H., Ryu J.H., Cho J.Y. (2017). Evaluation of soil pore-water salinity using a Decagon GS3 sensor in saline-alkali reclaimed tidal lands. Comput. Electron. Agric..

[13] Nie M., Zhang X., Wang J.Q., Jiang L.F., Yang J., Quan Z.X., Cui X.H., Fang C.M., Li B. (2009). Rhizosphere effects on soil bacterial abundance and diversity in the Yellow River Deltaic ecosystem as influenced by petroleum contamination and soil salinization. Soil Biol. Biochem..

[14] Wang Z., Lin K., Liu X. (2022). Distribution and pollution risk assessment of heavy metals in the surface sediment of the intertidal zones of the Yellow River Estuary, China. Mar. Pollut. Bull..

[15] Tavakkoli E., Rengasamy P., McDonald G.K. (2010). High concentrations of Na^+^ and Cl^−^ ions in soil solution have simultaneous detrimental effects on growth of faba bean under salinity stress. J. Exp. Bot..

[16] Zuffo A.M., Aguilera J.G., Silva F.C.D.S., Mezzomo R., Barrozo L.M., Steiner F., Oliveira B.R., Soto C.A.M. (2025). Multivariate Adaptability of Tropical Wheat Cultivars to Drought and Salinity Stresses. Plants.

[17] Lu L., Wu X., Tang Y., Zhu L., Hao Z., Zhang J., Li X., Shi J., Chen J., Cheng T. (2022). Halophyte *Nitraria billardieri CIPK25* promotes photosynthesis in *Arabidopsis* under salt stress. Front. Plant Sci..

[18] Yuan L., Gao Y., Cheng F., Du J., Hu Z., Yang X., Wang H., Kong X. (2022). The influence of oil exploitation on the degradation of vegetation: A case study in the Yellow River Delta Nature Reserve, China. Environ. Technol. Innov..

[19] Zhang X., Qi A., Wang P., Huang Q., Zhao T., Yan C., Yang L., Wang W. (2023). Spatial distribution, sources, air-soil exchange, and health risks of parent PAHs and derivative-alkylated PAHs in different functional areas of an Oilfield Area in the Yellow River Delta, North China. Toxics.

[20] Zhang X., Liu Z., Luc N.T., Yu Q., Liu X., Liang X. (2016). Impacts of soil petroleum contamination on nutrient release during litter decomposition of *Hippophae rhamnoides*. Environ. Sci. Process. Impacts.

[21] Haghollahi A., Fazaelipoor M.H., Schaffie M. (2016). The effect of soil type on the bioremediation of petroleum contaminated soils. J. Environ. Manag..

[22] Yuan L., Wu Y., Fan Q., Li P., Liang J., Liu Y., Ma R., Li R., Shi L. (2023). Remediating petroleum hydrocarbons in highly saline–alkali soils using three native plant species. J. Environ. Manag..

[23] Singh S., Prasad S.M. (2014). Growth, photosynthesis and oxidative responses of *Solanum melongena* L. seedlings to cadmium stress: Mechanism of toxicity amelioration by kinetin. Sci. Hortic..

[24] Liu S., Yang C., Xie W., Xia C., Fan P. (2012). The effects of cadmium on germination and seedling growth of *Suaeda salsa*. Procedia Environ. Sci..

[25] Xiong J., Shao X., Li N., Yuan H., Liu E., Wu M. (2024). Effects of land-use on soil C, N, and P stocks and stoichiometry in coastal wetlands dependent on soil depth and latitude. Catena.

[26] Hu Y., Yan T., Gao Z., Wang T., Lu X., Yang L., Shen L., Zhang Q., Hu J., Ren D. (2024). Appropriate Supply of Ammonium Nitrogen and Ammonium Nitrate Reduces Cadmium Content in Rice Seedlings by Inhibiting Cadmium Uptake and Transport. Rice Sci..

[27] Xiao Z., Duan C., Li S., Chen J., Peng C., Che R., Liu C., Huang Y., Mei R., Xu L. (2023). The microbial mechanisms by which long-term heavy metal contamination affects soil organic carbon levels. Chemosphere.

[28] Feng L., Xia J.B., Liu J.T., Song A.Y., Chen Y.P., Zhao X.M. (2021). Effects of mosaic biological soil crusts on vascular plant establishment in a coastal saline land of the Yellow River Delta, China. J. Plant Ecol..

[29] Moghaieb R.E.A., Saneoka H., Fujita K. (2004). Effect of salinity on osmotic adjustment, glycinebetaine accumulation and the betaine aldehyde dehydrogenase gene expression in two halophytic plants, *Salicornia europaea* and *Suaeda maritima*. Plant Sci..

[30] Xie T., Liu X., Sun T. (2011). The effects of groundwater table and flood irrigation strategies on soil water and salt dynamics and reed water use in the Yellow River Delta, China. Ecol. Model..

[31] Zhao Y., Li T., Liu J., Sun J., Zhang P. (2022). Ecological stoichiometry, salt ions and homeostasis characteristics of different types of halophytes and soils. Front. Plant Sci..

[32] Shang C., Wang L., Tian C., Song J. (2020). Heavy metal tolerance and potential for remediation of heavy metal-contaminated saline soils for the euhalophyte *Suaeda salsa*. Plant Signal. Behav..

[33] Mohammed H.A. (2020). The Valuable Impacts of Halophytic Genus Suaeda; Nutritional, Chemical, and Biological Values. Med. Chem..

[34] Qu F., Meng L., Xia J., Huang H., Zhan C., Li Y. (2021). Soil phosphorus fractions and distributions in estuarine wetlands with different climax vegetation covers in the Yellow River Delta. Ecol. Indic..

[35] Liu H., Huang X., Fan X., Wang Q., Liu Y., Wei H., He J. (2023). Phytoremediation of crude oil-contaminated sediment using *Suaeda heteroptera* enhanced by *Nereis succinea* and oil-degrading bacteria. Int. J. Phytoremediation.

[36] Yi L.P., Wang Z.W. (2017). Effects of different types of halophytes on the concentration of cadmium in coastal saline soil. Acta Ecol. Sin..

[37] Meng L., Qu F., Bi X., Xia J., Li Y., Wang X., Yu J. (2021). Elemental stoichiometry (C, N, P) of soil in the Yellow River Delta nature reserve: Understanding N and P status of soil in the coastal estuary. Sci. Total Environ..

[38] Huang Y., Hu X.Y., Cao K.K., Zhang M., Hu X.X., Wang Z.J. (2021). Interaction and Mechanism Between Conditioning Agents and Two Elements in the Soil Enriched with Phosphorus and Cadmium. Huan Jing Ke Xue.

[39] Li X., Liu X., Zhang Y., Liu J., Huang Y., Li J. (2024). Seasonal Effects of Constructed Wetlands on Water Quality Characteristics in Jinshan Lake: A Gate Dam Lake (Zhenjiang City, China). Biology.

[40] Lerdau M., Coley P.D. (2002). Benefits of the carbon-nutrient balance hypothesis. Oikos.

[41] Sardans J., Peñuelas J. (2012). The role of plants in the effects of global change on nutrient availability and stoichiometry in the plant-soil system. Plant Physiol..

[42] Su Z., Su B., Mao S., Shangguan Z. (2023). Leaf C: N: P stoichiometric homeostasis of a *Robinia pseudoacacia* plantation on the Loess Plateau. J. For. Res..

[43] Li Y., Rahman S.U., Qiu Z., Shahzad S.M., Nawaz M.F., Huang J., Naveed S., Li L., Wang X., Cheng H. (2023). Toxic effects of cadmium on the physiological and biochemical attributes of plants, and phytoremediation strategies: A review. Environ. Pollut..

[44] Zhang W., Liu W., Xu M., Deng J., Han X., Yang G., Feng Y., Ren G. (2019). Response of forest growth to C:N:P stoichiometry in plants and soils during *Robinia pseudoacacia* afforestation on the Loess Plateau, China. Geoderma.

[45] Jing J., Zhang F., Rengel Z., Shen J. (2012). Localized fertilization with P plus N elicits an ammonium-dependent enhancement of maize root growth and nutrient uptake. Field Crops Res..

[46] Qu F., Shao H., Meng L., Yu J., Xia J., Sun J., Li Y. (2018). Forms and vertical distributions of soil phosphorus in newly formed coastal wetlands in the Yellow River Delta estuary. Land Degrad. Dev..

[47] Brinch U.C., Ekelund F., Jacobsen C.S. (2002). Method for spiking soil samples with organic compounds. Appl. Environ. Microbiol..

[48] Nie M., Wang Y., Yu J., Xiao M., Jiang L., Yang J., Fang C., Chen J., Li B. (2011). Understanding plant-microbe interactions for phytoremediation of petroleum-polluted soil. PLoS ONE.

[49] Ministry of Ecology and Environment of China, State Administration for Market Regulation of China (2018). Soil Environmental Quality Risk Control Standard for Soil Contamination of Agricultural Land.

[50] Ministry of Environmental Protection (2014). Technical Guidelines for Risk Assessment of Contaminated Sites.

[51] Yu W., Wu W., Zhang N., Wang L., Wang Y., Wang B., Lan Q., Wang Y. (2022). Research Advances on Molecular Mechanism of Salt Tolerance in *Suaeda*. Biology.

[52] Liu S., Zhu L., Jiang W., Qin J., Lee H.S. (2021). Research on the effects of soil petroleum pollution concentration on the diversity of natural plant communities along the coastline of Jiaozhou bay. Environ. Res..

[53] Chigbo C., Batty L. (2013). Phytoremediation potential of Brassica juncea in Cu-pyrene co-contaminated soil: Comparing freshly spiked soil with aged soil. J. Environ. Manag..

[54] Shidan B. (2000). Soil Agrochemical Analysis.

